# Estimation of the basic reproduction number of Alpha and Delta variants of COVID-19 pandemic in Iran

**DOI:** 10.1371/journal.pone.0265489

**Published:** 2022-05-17

**Authors:** Farnaz Sheikhi, Negar Yousefian, Pardis Tehranipoor, Zahra Kowsari

**Affiliations:** Faculty of Computer Engineering, K. N. Toosi University of Technology, Tehran, Iran; Konkuk University, KOREA, REPUBLIC OF

## Abstract

Estimating the basic reproduction number of a pandemic and the changes that appear on this value over time provide a good understanding of the contagious nature of the virus and efficiency of the controlling strategies. In this paper, we focus on studying the basic reproduction number (*R*_0_) for two important variants of COVID-19 pandemic in Iran: Alpha and Delta variants. We use four different methods, three statistical models and one mathematical model, to compute *R*_0_: Exponential Growth Rate (EGR), Maximum Likelihood (ML), Sequential Bayesian (SB), and time-dependent SIR model. Alpha variant of COVID-19 was active in Iran from March 10, 2021 until June 10, 2021. Our computations indicate that total *R*_0_ of this variant according to EGR, ML, SB, and SIR model is respectively 0.9999 (95% CI: 0.9994-1), 1.046 (95% CI: 1.044-1.049), 1.06 (95% CI: 1.03-1.08), and 2.79 (95% CI: 2.77-2.81) in the whole active time interval. Moreover, during the time interval from April 3, 2021 to April 9, 2021 in which this variant was in its exponential growth in Iran, *R*_0_ of Alpha variant in Iran according to SB, EGR, ML, and SIR model is respectively 2.26 (95% CI: 2.04-2.49), 2.64 (95% CI: 2.58-2.7), 11.38 (95% CI: 11.28-11.48), and 12.13 (95% CI: 12.12-12.14). Delta variant was active in Iran during the time interval from June 22, 2021 until September 22, 2021. Our computations show that during the time interval from July 3, 2021 to July 8, 2021 in which this variant was in its exponential growth in Iran, *R*_0_ of Delta variant in Iran according to SB, EGR, ML, and SIR model is respectively 3 (95% CI: 2.34-3.66), 3.1 (95% CI: 3.02-3.17), 12 (95% CI: 11.89-12.12), and 23.3 (95% CI: 23.19-23.41). Further, total *R*_0_ of Delta variant in Iran in the whole active time interval according to EGR, ML, SB, and SIR model is respectively 1.042 (95% CI: 1.04-1.043), 1.053 (95% CI: 1.051-1.055), 0.79 (95% CI: 0.63-0.95), and 5.65 (95% CI: 5.6-5.7). As the results show Delta variant was more severe than Alpha variant in Iran. Chasing the changes in *R*_0_ during each variant shows that the controlling strategies applied were effective in controlling the virus spread.

## Introduction

After the first appearance of a novel type of pneumonia in December 2019 in Wuhan, Hubei Province of China, a new type of coronavirus, initially named 2019-nCoV on January 7, 2020, was identified as the cause of the disease [1, 2]. Examining the genome sequence of this virus declared a high similarity to Severe Acute Respiratory Syndrome-related coronavirus (SARS-CoV) that swept China in 2003. Thus, on February 11, 2020, the International Committee on Taxonomy of Viruses (ICTV) renamed 2019-nCoV to Severe Acute Respiratory Syndrome Coronavirus 2 (SARS-CoV-2) [3]. This virus got briefly referred as COVID-19 then by the World Health Organization (WHO) [4]. The most common symptoms of COVID-19 include fever, cough, fatigue, and dyspnea [5, 6]. COVID-19 started to rapidly spread not only in China, but also in all over the world. Unfortunately, in less than four months after the initiation of this virus, more than 118,000 new cases were identified in 114 different countries. Acceleration and high volume transmission of the infection of COVID-19 made this virus classified as a pandemic on March 11, 2020, by WHO [7]. Since then several different mutations of COVID-19, *Alpha, Beta, Gamma, Delta*, and newly appeared *Omicron* variants, have been emerged with difference in severity and transmission rate of this virus [8, 9]. Preventive strategies such as social distancing, mask wearing, and regular hand-washing on one side, and strengthening the immune system by following a balanced diet enriched with adequate amount of protein, vitamins, and minerals on the other side play a crucial role against COVID-19 [6, 10, 11]. Further, high rate of COVID-19 global vaccination has a vital impact on mitigating COVID-19 [12, 13]. Hopefully, several COVID-19 vaccines have been authorized so far, and some new ones are under different steps of trial [12–14]. Although efficiency of vaccination is not ignorable, sufficiency of primary vaccination is debatable. Since vaccine-induced protection from COVID-19 changes variant by variant, boosting may be ultimately needed [15]. Therefore, studying epidemiologic characteristics of variants of COVID-19 is crucial.

The variant of B.1.1.7 which is also known as Alpha variant was first observed in the south of England [16]. The patients infected by B.1.1.7 had more cough, sore throat, myalgia, and fatigue. Loss of sense of smell and taste were less reported among infections of this variant [17]. Combining several behavioral and epidemiological data sources with statistical and dynamic modeling have indicated that Alpha variant of COVID-19 pandemic is approximately 43% to 100% more transmissible than the previous infections [18]. On December 18, 2020, at the same time as the designation of Alpha variant, African authorities identified a new variant of COVID-19, called B.1.351 or Beta variant, which was swiftly infecting people [19]. Study of Beta variant showed more infections in elderly people, and increase in the number of hospitalizations and deaths compared to Alpha variant [20]. Another variant of COVID-19 pandemic found in Brazil on January 21, 2021, was called P.1 or Gamma variant. Mutations in Gamma variant caused reinfection to the virus in spite of existent antibodies [21]. Following that, in April 2021, the variant of B.1.617.2 or Delta variant was first identified in India [9]. The most common symptoms in the individuals with Delta variant were headache, sore throat, and a runny nose [22]. It is reported that one dose of COVID-19 vaccine does not provide adequate resistance against Delta variant [8]. Due to faster transmission rate and less effectiveness of known public health treatments in this variant, WHO considered Delta variant as a Variant of Concern (VOC) on May 11, 2021 [9]. The number of total infections classified by variants of COVID-19 and countries are presented in Table 1.

**Table 1:** Number of COVID-19 infections by October 18, 2021, classified by variants and countries.

Country	Alpha Variant	Beta Variant	Gamma Variant	Delta Variant
**Italy**	25,979 [23]	128 [24]	2,585 [25]	22,588 [26]
**India**	4,184 [23]	240 [24]	5 [25]	36,509 [26]
**SriLanka**	430 [23]	6 [24]	unknown	730 [26]
**Turkey**	1,916 [23]	502 [24]	169 [25]	45,200 [26]
**USA**	232,486 [23]	2,998 [24]	27,870 [25]	588,640 [26]

In Iran, the first case of COVID-19 infection was noticed in Qom on February 19, 2020 [27]. Since then, the number of confirmed cases has risen steeply so that by October 11, 2021, the cumulative total number of confirmed cases and deaths due to COVID-19 in Iran was respectively 5,716,394 and 122,868 [28]. Table 2 provides a detailed description about the total number of infections and deaths in two important variants of COVID-19 pandemic, Alpha and Delta variant, in Iran. The data is collected form Iranian Students News Agency (ISNA) [29]. Although acceleration of vaccination plays a vital role in deceleration of the infection, the impact of vaccination is not similar against different variants of COVID-19 pandemic. Variant by variant, COVID-19 virus empowers. The soonest this pandemic is closed, the less costs are forced on societies. Much is still unknown about the nature of this virus. Therefore, study of COVID-19 properties, such as the transmission rate of different variants can improve knowledge about nature of the virus, and can help societies with effective strategies to slow down the speed of spread or even stop this pandemic [30].

**Table 2:** The number of total cases and deaths in Alpha and Delta variants of COVID-19 in Iran.

Variant	Total Cases	Total Deaths
**Alpha variant**	1,296,553	20,929
**Delta variant**	2,596,064	39,787

One of the tools that can be utilized to evaluate the dissemination and transmission rate of a pandemic in distinctive periods is the *basic reproduction number*, denoted by *R*_0_. The basic reproduction number is defined as the average number of secondary cases directly generated by an individual infection [31]. It assumes that the entire population is susceptible against the infection, which is true at the beginning of the virus spread. Thus, the basic reproduction number describes the power of virus transmission under no external control. External controls such as city lockdown, isolation of confirmed cases, and traffic control can play a significant role in decreasing the transmissibility of the virus [32]. When *R*_0_ > 1, the virus is spreading. *R*_0_ becoming less and less, shows the effectiveness of the external controls, and when *R*_0_ < 1, it means the pandemic is over [33]. Estimating *R*_0_ for a long-running outbreak, such as COVID-19 pandemic, provides a better recognition of nature of the virus and is helpful for establishing and evaluating controlling strategies for the virus spread. Table 3 specifies *R*_0_ for several epidemics so far. Moreover, Table 4 estimates *R*_0_ of the ongoing COVID-19 pandemic during specific time intervals in several countries.

**Table 3:** Estimated *R*_0_ for various epidemics.

Disease	Location	Date	*R*_0_	Reference
**Measles**	Ghana	1960–68	14.5	[34]
**SARS**	29 countries	2002–03	3.5	[35]
**H1N1 influenza**	South Africa	2009	1.33	[36]
**Ebola**	Guinea	2014	1.51	[37]
**Zika**	South America	2015–16	2.06	[38]

**Table 4:** Estimated *R*_0_ for COVID-19 in different countries.

Country	Time Span	*R*_0_	Reference
**Italy**	February 23-March 9, 2020	3.17–3.38	[39]
**India**	March 2-May 7, 2020	1.379	[40]
**Iran**	February 19-March 11, 2020	3.23–4.70	[41]
**Sri Lank**	March 11-April 30, 2020	0.93–1.23	[42]
**Turkey**	November 16-December 9, 2020	1.38	[43]

There are few works studying *R*_0_ for different variants of COVID-19 pandemic in different countries as far as we are aware are, although the general knowledge is Delta variant is more contagious than the previous variants of COVID-19 [44]. A study in Scotland reported that Delta variant doubled the risk of hospitalization compared to Alpha variant of COVID-19 pandemic especially for unvaccinated people, indicating that Delta variant caused more severe infections [45]. Hence, in this paper we focus on providing a comparative study of *R*_0_ of Alpha and Delta variants of COVID-19 pandemic in Iran.

## Data source

**Fig 1:**
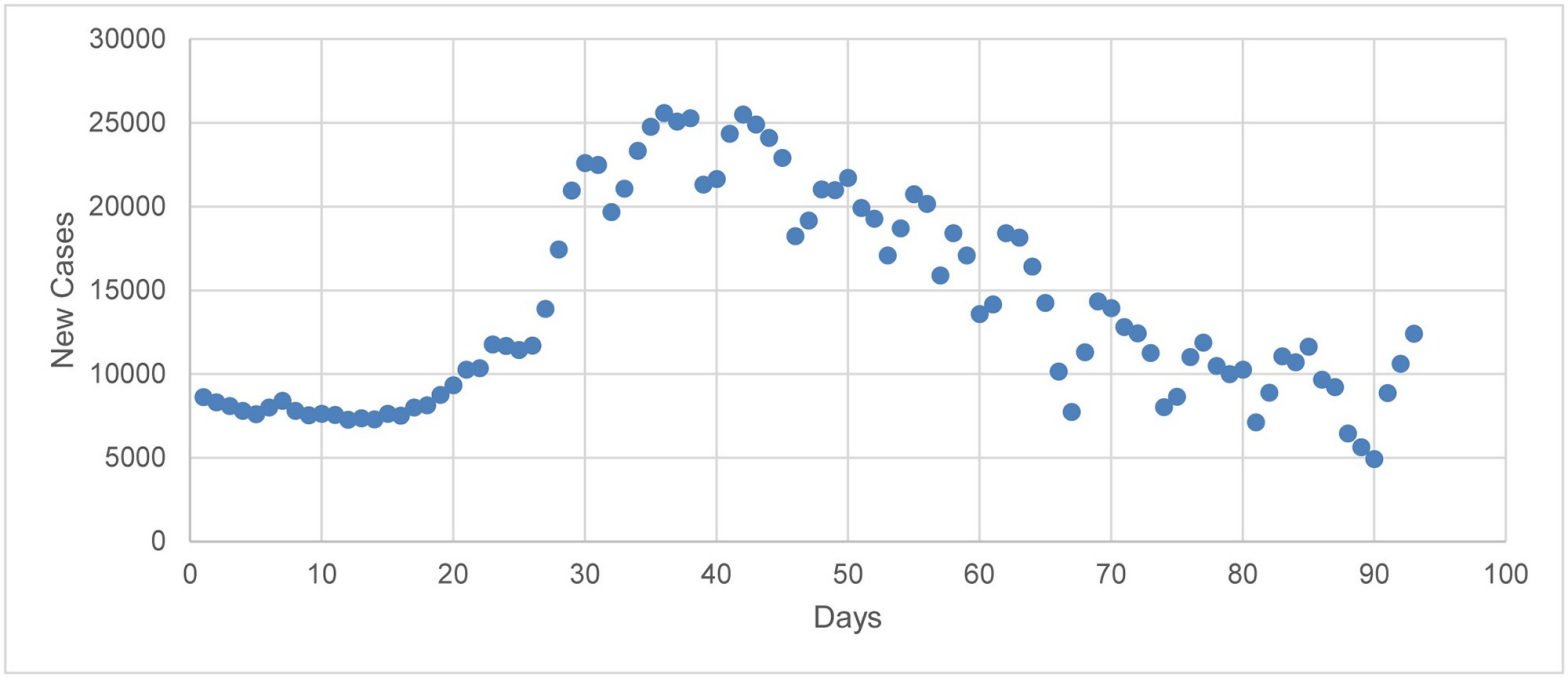
The number of daily infections for Alpha variant in Iran.

**Fig 2:**
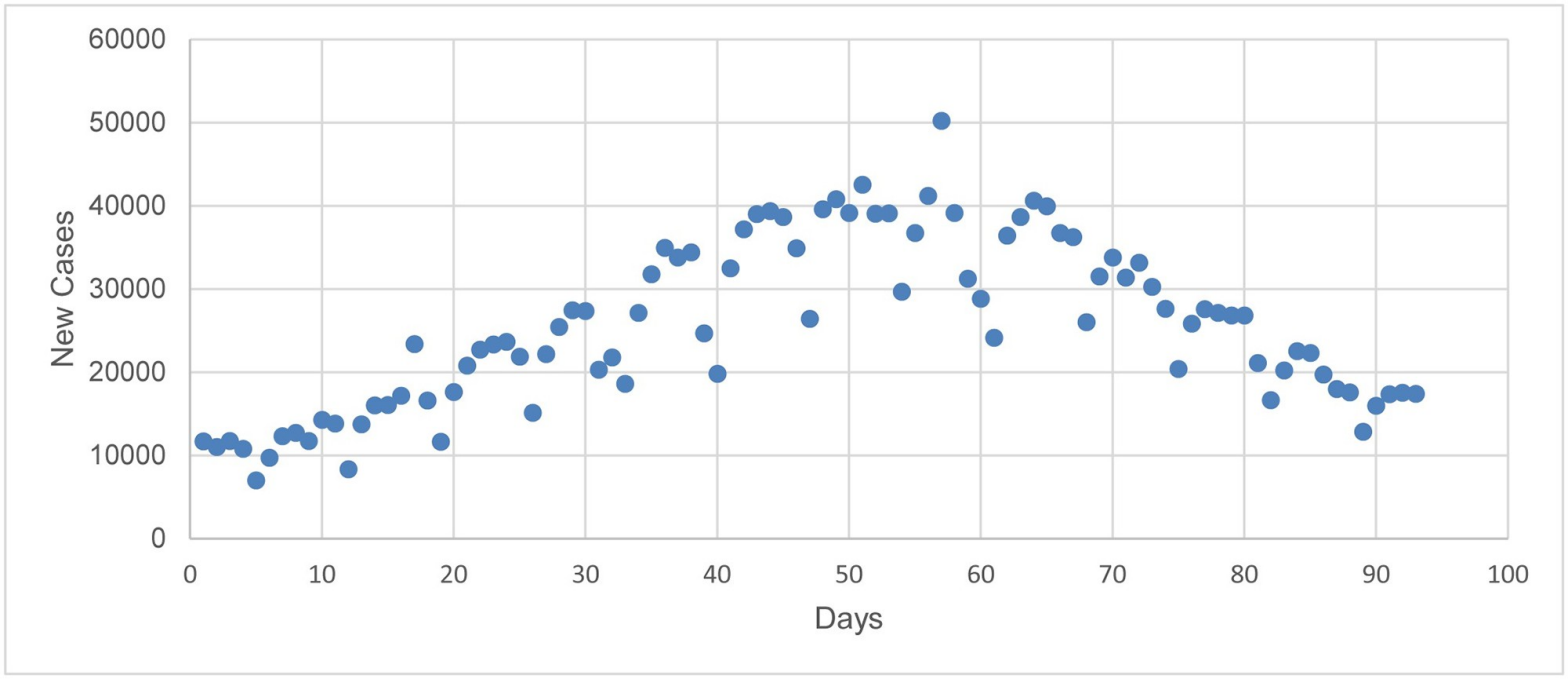
The number of daily infections for Delta variant in Iran.

For our analysis of the basic reproduction number (*R*_0_) of variants of COVID-19 pandemic in Iran, we have collected our data set from Iranian Students News Agency (ISNA), daily outbreak news [29]. We have collected data including the number of infected cases, the number of recovered cases, and the number of deaths for two different time intervals: first from March 10, 2021 to June 10, 2021 (Alpha variant) and second from June 22, 2021 to September 22, 2021 (Delta variant). Figs 1 and 2 illustrate the diagram of daily infections in Iran classified by Alpha and Delta variants.

## Methods

There are various methods used for estimating *R*_0_, ranging from the epidemiological compartmental models such as SIR to a variety of statistical methods. Due to different nature of each method, the values produced by these methods can not be completely comparable with each other. However, tracking *R*_0_ value of each method can provide a good understanding of the virus behavior. In this paper, we use time-dependent SIR model and the three most famous statistical methods (maximum likelihood, exponential growth rate-based method, and sequential Baysian method) for estimating *R*_0_ of Alpha and Delta variants of COVID-19 in Iran. These methods are explained in details in the following.

### Time-dependent SIR model

Time-dependent SIR model is a simple and at the same time an efficient compartmental epidemiological model for infectious diseases [33]. In this model, the population is divided into three categories: Susceptible (*S*), Infectious (*I*), and Recovered (*R*). At the beginning of a pandemic, all population is in the state *S*. Then, they move to the state *I* with the rate of *β*, and afterwards to the state *R* with the rate of *γ*. Hence, SIR model has two time-varying variables: the transmission rate *β* and the recovering rate *γ*. The basic reproduction number in this model is defined as *β*(*t*)/*γ*(*t*) since an infected person will be recovered in 1/*γ* days (on average), and he/she is in contact with *β* (on average) people during the infectious period. Thus, in time-dependent SIR model, *S*(*t*), *X*(*t*) and *R*(*t*) respectively indicates the number of susceptible, the number of infected, and the number of recovered people at time *t*. In order to compute *R*_0_ in this model, *β*(*t*) and *γ*(*t*) should be calculated. This model follows the following discrete difference equations [46]:

$$\begin{array}{c} S\left(t+1\right)-S\left(t\right)=\frac{-\beta (t)S(t)X(t)}{n} \end{array}$$ (1)

$$\begin{array}{c} X\left(t+1\right)-X\left(t\right)=\frac{\beta (t)S(t)X(t)}{n}-\gamma (t)X(t) \end{array}$$ (2)

$$\begin{array}{c} R(t+1)-R(t)=\gamma (t)X(t) \end{array}$$ (3)

The parameter *n* is the total number of population. At the beginning of a pandemic, most of the population are in the susceptible state; as a result, we assume *S*(*t*) ≃ *n*, *t* ≥ 0. So, we can simplify Eqs (1) and (2) as follows:

$$\begin{array}{c} S(t+1)-S(t)=-\beta (t)S(t)X(t) \end{array}$$ (4)

$$\begin{array}{c} X(t+1)-X(t)=\beta (t)S(t)X(t)-\gamma (t)X(t) \end{array}$$ (5)

From the difference equations above, *β*(*t*) and *γ*(*t*) can be easily computed as

$$\begin{array}{c} \beta \left(t\right)=\frac{\left[X(t+1)-X(t)]+[R(t+1)-R(t)\right]}{X(t)} \end{array}$$ (6)

$$\begin{array}{c} \gamma \left(t\right)=\frac{R(t+1)-R(t)}{X(t)} \end{array}$$ (7)

Now, fitting the data set to these simplified equations leads to estimating *R*_0_ for the corresponding pandemic.

### Maximum Likelihood method

Using Maximum Likelihood method (ML) for estimating *R*_0_ was introduced by White and Pagano [47]. This method assumes that a primary case generates the secondary cases according to a Poisson distribution with mean *R*_0_. In this method, *N*_0_, …, *N*_*t*_ demonstrate the incident cases, and *W* represents the generation time distribution. Therefore, *R*_0_ can be estimated by maximizing the log-likelihood as follows.

$$\begin{array}{c} LL\left(R_{0}\right)=\sum_{t=1}^{T}\text{log}\;\frac{e^{-\mu_{t}}\mu_{t}^{N_{t}}}{N_{t}!} \end{array}$$ (8)

Where

$$\begin{array}{c} \mu_{t}=R_{0}\sum_{i=1}^{t}N_{t-i}W_{i} \end{array}$$ (9)

The likelihood should be computed in a period of exponential growth. It is noteworthy to mention that maximum likelihood method has some assumptions and if any infraction happens, the result alters. These assumptions are as follows: No data should be missing and the population has to be uniformly mixed.

### Exponential Growth Rate-based method

The method of Exponential Growth Rate (EGR) for calculating *R*_0_ was introduced by Wallinga and Lipsitch [48]. In this method, first a time span in which the epidemic curve has an exponential growth is selected, where *r* indicates the growth rate (changes in the number of new incidents), and is calculated by Poisson regression. Then, *R*_0_ is estimated using the moment generating function of the generating time distribution, *M*, which is:

$$\begin{array}{c} R_{0}=\frac{1}{M(-r)} \end{array}$$ (10)

### Sequential Bayesian method

Bayesian time-dependant method proposed by Bettencourt and Ribeiro [49] calculates *R*_0_ using Poisson distribution in a time period with exponential growth. This method works by starting with a slightly informative prior on *R*_0_ and updates it sequentially. That is why the method is also referred as Sequential Bayesian (SB). Let *N*_*t*+1_ be the number of cases in time *t* + 1 with an approximate Poisson distribution where *N*(*t*)*e*^(*γ*(*R*−1))^ is the mean of the distribution and $\frac{1}{\gamma }$ specifies the average of the infection period. In this method, *P*(*R*_0_) is the prior, which captures information of the distribution of *R*_0_. The prior distribution of each day demonstrates the posterior distribution of the day before and will be recalculated by:

$$\begin{array}{c} P\left(R_{0}|N_{0},\ldots ,N_{t+1}\right)=\frac{P\left(N_{t+1}|R_{0},N_{0},\ldots ,N_{t}\right)P\left(R_{0}|N_{0},\ldots N_{t}\right)}{P(N_{0},\ldots N_{t+1})} \end{array}$$ (11)

As a limitation of this method is the fact that this method can not be used for data sets with no new infection in some time intervals, since it leads to a Poisson mean of zero.

## Results

All the the statical analysis were performed using software R version 4.1.1 and the *R*_0_ package [50]. Since we did not have access to the clinical COVID-19 data, the parameters of the serial interval distribution were not computed directly. And according to the fact that nature of COVID-19 virus is the same worldwide, we assume the fitting model is the same as the models already observed in COVID-19 pandemic worldwide, which is a Gamma distribution with a mean of 7.5 days and a standard deviation of 3.4 days [51]. Then, the basic reproduction number (*R*_0_) of Alpha and Delta variants of COVID-19 pandemic in Iran is estimated by using four different methods: EGR, ML, SB, and SIR. We have used two time intervals for each variant: time interval with exponential growth and the total active time interval. Results of computing *R*_0_ in the time spans with exponential growth are classified in Table 5.

**Table 5:** Estimated *R*_0_ of Alpha and Delta variants of COVID-19 pandemic in Iran in a time span of exponential growth.

Variant	Time Span	EGR	ML	SB	SIR
**Alpha**	April 3-April 9, 2021	2.6422 (95% CI: 2.58–2.7)	11.3811 (95% CI: 11.28–11.48)	2.2680 (95% CI: 2.06–2.46)	12.1317 (95% CI: 12.12–12.14)
**Delta**	July 3-July 8, 2021	3.1002 (95% CI: 3.02–3.17)	12.0089 (95% CI: 11.89–12.12)	3.0060 (95% CI: 2.34–3.66)	23.3021 (95% CI: 23.19–23.41)

Considering the estimated *R*_0_ for the total time intervals in which Alpha and Delta variants were active in Iran as shown in Table 6, proves higher contagion of Delta variant than Alpha variant in Iran. Further, in the time span that the number of infections increased exponentially, the results showed more transmissibility of each variant. Moreover, *R*_0_ for the total time interval of Alpha and Delta in Iran is respectively less than *R*_0_ of each of these variants in the time span with the exponential growth. This indicates that the controlling strategies in both variants were effective to control the virus spread. Table 6 represents the details.

**Table 6:** Estimated total *R*_0_ of Alpha and Delta variants in Iran.

Variant	Total Time Interval	EGR	ML	SB	SIR
**Alpha**	March 10-June 10, 2021	0.9999 (95% CI: 0.99941–1.00043)	1.0468 (95% CI: 1.044–1.049)	1.0613 (95% CI: 1.034–1.089)	2.7949 (95% CI: 2.77–2.81)
**Delta**	June 22-September 22, 2021	1.0426 (95% CI: 1.042–1.043)	1.0538 (95% CI: 1.051–1.055)	0.7970 (95% CI: 0.63–0.95)	5.6577(95% CI: 5.6–5.7)

## Discussion

The results of estimating the basic reproduction number for each COVID-19’s variants show that Delta variant has more infectivity compared to Alpha variant, as confirmed by health organizations. However, the strategies that have been applied, have had positive impacts on controlling the virus spread of each variant.

Of the limitations of this study can be the fact that the reported number of infections is based on COVID-19 RT-PCR tests with positive results. However, this test can have a high rate of false-negative result [52]. Hence, due to failure of RT-PCR tests in precisely detecting COVID-19 and its variants, the number of infected people is not completely accurate. Therefore, using complementary tests may result in a more accurate number of infections. Moreover, the vaccination rate has had a powerful effect on controlling the spread of COVID-19, which is not considered in the models provided for estimating the basic reproduction number. Thus, further studies should take into account all contributing factors to the basic reproduction number of COVID-19 pandemic.

## Conclusion

As a conclusion, *R*_0_ of Alpha variant of COVID-19 pandemic in Iran is estimated between 2.26–11.38 by the statistical methods, and 12.13 by time dependent SIR model in the time interval with exponential growth. Further, *R*_0_ of this variant in the total time interval of activation is estimated between 0.9–1.6 by the statistical methods, and 2.79 by time-dependent SIR model. For Delta variant of COVID-19 pandemic in Iran, estimated *R*_0_ is in range 3.0–12.0 by the statistical methods, and 23.3 by time-dependent SIR model in the time interval with exponential growth. And in the total time interval of activation of Delta variant in Iran, the estimated *R*_0_ is in range 0.797–1.05 by the statistical methods, and 5.65 by time-dependent SIR model. The results specify that the nature of Delta variant is more contagious than Alpha variant in Iran. Decrease in *R*_0_ in the total time interval of each variant indicates the positive effects of the controlling strategies such as traffic restrictions, distance working, school closures, and cancelation of mass gatherings in reducing the virus spread. Although the nature of Delta variant in Iran is more contagious that Alpha variant, as Table 2 confirms doubling of the number of cases in Delta variant in Iran, the proximity of *R*_0_ for the total active time of Alpha and Delta variants in Iran confirms the positive effect of accelerating the vaccination from the beginning of the Delta variant in Iran [53].

## Supporting information

S1 File Alpha dataset. The dataset that was used for calculating Reproduction number for the Alpha variant.
(TXT)

S2 File Delta dataset. The dataset that was used for calculating Reproduction number for the Delta variant.
(TXT)
